# Nanoparticles and Their Antibacterial Application in Endodontics

**DOI:** 10.3390/antibiotics12121690

**Published:** 2023-12-01

**Authors:** Nicoletta Capuano, Alessandra Amato, Federica Dell’Annunziata, Francesco Giordano, Veronica Folliero, Federica Di Spirito, Pragati Rajendra More, Anna De Filippis, Stefano Martina, Massimo Amato, Massimiliano Galdiero, Alfredo Iandolo, Gianluigi Franci

**Affiliations:** 1 Department of Medicine, Surgery and Dentistry “Scuola Medica Salernitana”, University of Salerno, 84081 Baronissi, Italy; capuanonicoletta95@gmail.com (N.C.); federica.dellannunziata@unicampania.it (F.D.); frgiordano@unisa.it (F.G.); vfolliero@unisa.it (V.F.); fdispirito@unisa.it (F.D.S.); smartina@unisa.it (S.M.); mamato@unisa.it (M.A.); 2 Department of Neuroscience, Reproductive Science and Dentistry, University of Naples Federico II, 80138 Naples, Italy; aaleamato@gmail.com; 3 Department of Experimental Medicine, University of Campania “Luigi Vanvitelli”, 80138 Naples, Italy; pragatimore98@gmail.com (P.R.M.); anna.defilippis@unicampania.it (A.D.F.); massimiliano.galdiero@unicampania.it (M.G.); 4 Complex Operative Unity of Virology and Microbiology, University Hospital of Campania “Luigi Vanvitelli”, 80138 Naples, Italy

**Keywords:** nanotechnology, infection, dentistry, cleaning, root canal, biofilm matrix

## Abstract

Root canal treatment represents a significant challenge as current cleaning and disinfection methodologies fail to remove persistent bacterial biofilms within the intricate anatomical structures. Recently, the field of nanotechnology has emerged as a promising frontier with numerous biomedical applications. Among the most notable contributions of nanotechnology are nanoparticles, which possess antimicrobial, antifungal, and antiviral properties. Nanoparticles cause the destructuring of bacterial walls, increasing the permeability of the cell membrane, stimulating the generation of reactive oxygen species, and interrupting the replication of deoxyribonucleic acid through the controlled release of ions. Thus, they could revolutionize endodontics, obtaining superior results and guaranteeing a promising short- and long-term prognosis. Therefore, chitosan, silver, graphene, poly(lactic) co-glycolic acid, bioactive glass, mesoporous calcium silicate, hydroxyapatite, zirconia, glucose oxidase magnetic, copper, and zinc oxide nanoparticles in endodontic therapy have been investigated in the present review. The diversified antimicrobial mechanisms of action, the numerous applications, and the high degree of clinical safety could encourage the scientific community to adopt nanoparticles as potential drugs for the treatment of endodontic diseases, overcoming the limitations related to antibiotic resistance and eradication of the biofilm.

## 1. Introduction

**Figure 1:**
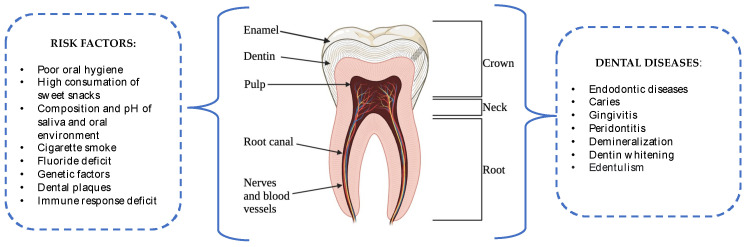
Schematic representation of tooth structure with common dental pathologies and related risk factors. Image created with BioRender.com (accessed on 10 August 2023).

Dental diseases represent a serious threat to human health, affecting approximately 3.5 million people each year [1]. Pathologies affecting teeth are the consequence of several factors, including (i) the oral microbiota; (ii) the genetic component of the host; (iii) lifestyle; (iv) the oral microenvironment, and so forth (Figure 1) [2]. Among the common diseases are endodontic ones, which occur when the dental pulp (odontoblasts, fibroblasts, undifferentiated mesenchymal cells, blood vessels, and nerves in the center of the tooth) undergoes infection through opportunistic pathogens and/or active inflammatory responses [3]. This has led the scientific community to direct its efforts towards the preventive and curative research of endodontists. Endodontic (root canal) treatment is a critical procedure aimed at removing damaged tissues and bacteria from the complex three-dimensional (3D) endodontic system [4]. One pivotal step in this treatment is the cleaning and shaping phase, involving the elimination of bacterial plaque from the anatomical regions and the instrumented portion of the root canal system [5]. To ensure effective cleaning, an active 3D approach is employed, allowing the irrigants into deeper areas to target pulp tissue and microbes in the most complex endodontic anatomies [6]. However, certain anatomical regions remain concealed and untreated during the mechanical shaping stage, leading to the accumulation of residual biofilms that might become a potential source of infection, jeopardizing the treatment prognosis [7]. Therefore, the elimination of these bacterial communities and their biofilms should be the first goal of these therapies to avoid the danger of re-infection [8]. Furthermore, the available antibacterial drugs have many difficulties penetrating dense extracellular matrix due to the different hydrophobic/hydrophilic properties and the size of the molecules [9].

**Figure 2:**
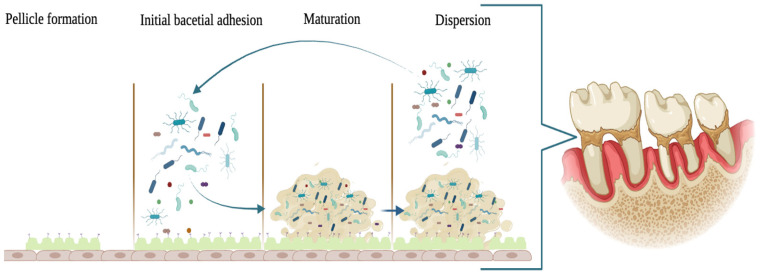
The oral biofilm formation stages: (1) salivary pellicle formation on the tooth surface; (2) adhesion of colonizing bacteria to the tooth through recognition of proteins present in the salivary pellicle; (3) biofilm formation and maturation; and (4) dispersion of planktonic bacteria from the matrix for new colonization. Image created with BioRender.com (accessed on 10 August 2023).

A significant challenge in root canal cleaning is addressing the dentinal tubules, microscopic structures easily inhabited by microorganisms that serve as potential sources of reinfection [10]. Following the chemo-mechanical preparation, a 1–2 µm thick smear layer is created, covering all the root canal walls. This smear layer contains inorganic dentin debris, organic substances, fragments of odontoblastic processes, bacteria, and necrotic pulp tissues, creating a haven for the remains of necrotic pulp tissues and biofilms [11]. The presence of the smear layer can inhibit the penetration of root canal irrigation solutions and intra-canal medications inside dentinal tubules, potentially affecting the bond strength of filling materials to the canal walls [12]. Mechanical manuals or rotary files alone are insufficient for eliminating bacteria from the root canal complex space. During the mechanical instrumentation phase, irrigating solutions play a vital role in purging debris, serving as a lubricant, and dissolving organic and inorganic tissue while providing a satisfactory antimicrobial effect [13]. Among these solutions, sodium hypochlorite (NaOCl) is widely favored due to its remarkable antimicrobial properties. NaOCl acts as an antiseptic, generating hypochlorous acid and releasing chlorine, an extremely active bactericide. Additionally, free chlorine in NaOCl can dissolve necrotic pulp tissue by disintegrating proteins into amino acids [14]. The failure of root canal treatment can occur when microbes persist in the root canal system [15]. Bacteria can survive in hostile circumstances, infiltrating dentinal tubules along the canal walls and forming biofilms (Figure 2) [7]. In this form, bacteria exhibit greater resistance to chemotherapeutic materials, medications, or even sealers compared to planktonic forms. The long- and short-term success of root canal treatment is directly linked to the persistence of the bacterial load at the end of therapy, before obturation. Consequently, enhancing the efficacy of disinfection protocols in endodontic therapy is crucial [16]. Currently, all these root canal irrigants and medications have not shown the ability to completely eradicate pathogens, necessitating alternative strategies to overcome this limitation.

Recently, nanotechnologies have emerged as an innovative solution in the treatment of dental infections through the synthesis of nanoparticles with antibacterial efficacy. Nanoparticles can be synthesized biologically or chemically and have been increasingly used in various biomedical and pharmaceutical industries due to their unique properties [17]. According to the European Commission’s Counsel, “nanomaterials” refer to natural, supplementary, or artificial materials holding particles, which can be unbound, combined, or agglomerated. For at least 50% of the particles in the size distribution, one or more external dimensions range between 1 and 100 nm [18]. Among the various nanomaterials, silver nanoparticles (AgNPs) have found extensive use due to their potent antimicrobial properties, capable of killing multi-drug-resistant microorganisms [19]. The small size of AgNPs allows them to penetrate cell membranes, damaging DNA and disintegrating biofilms. Currently, nanoparticles, especially AgNPs, are considered an alternative antibiotic strategy. However, it is essential to consider the size-dependent toxicity of nanoparticles, as smaller sizes may result in higher toxicity due to increased reactivity and ion release in cells [20]. Incorporating nanoparticles into biomaterials used in endodontics has shown promising results in augmenting antimicrobial properties [21,22,23,24,25,26]. This innovative approach opens up new possibilities for the prevention and treatment of dental infections. By linking nanoparticles with polymers or coating them with biomaterials, their antimicrobial effectiveness can be enhanced. In this review, we explore the impact of incorporating nanoparticles into different biomaterials used in endodontics.

## 2. Methodology

**Figure 3:**
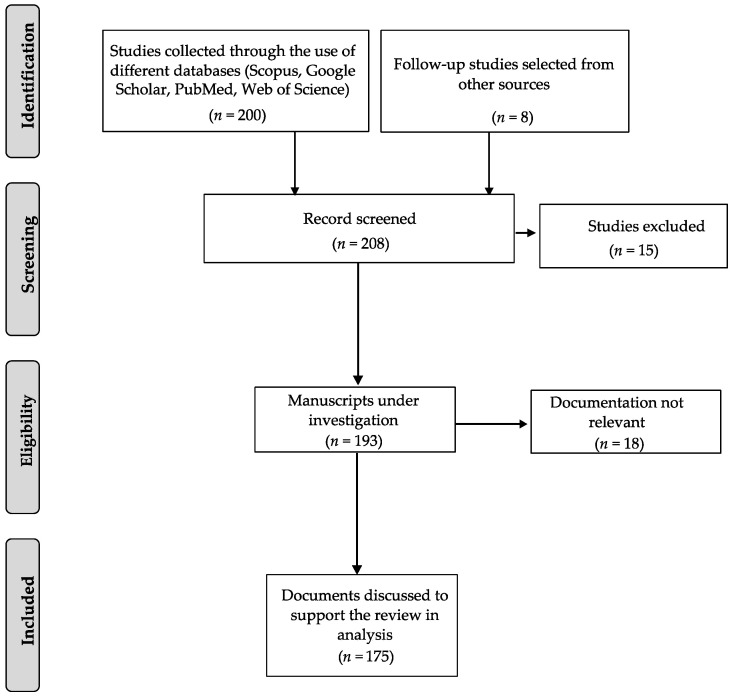
PRISMA flowchart for the selection of studies discussed in the present review [27].

The present review comprises a comprehensive collection of articles focusing on the innovative application of nanoparticles in endodontic therapy. The relevant literature was extensively searched for and gathered through various electronic databases, such as PubMed and Google Scholar, followed by Scopus and Web of Science. The bibliographic research utilized specific keywords, including “nanoparticles”, “endodontic therapy”, “root canal”, “root canal treatment”, “antibacterial nanoparticles”, and “biofilm and nanoparticles.” Articles were selected based on two primary criteria: (i) recent studies related to alternative endodontic therapies, and (ii) exploration of nanoparticles’ applications across diverse medical fields. Following these criteria, 187 articles published until now were carefully chosen, summarized, and critically analyzed to provide a cohesive and informative review. The PRISMA flowchart for study selection is depicted in Figure 3. In the subsequent sections, a thorough discussion of all available articles concerning nanoparticles and endodontic therapies will be presented.

## 3. Mechanism of Action of Nanoparticles

**Figure 4:**
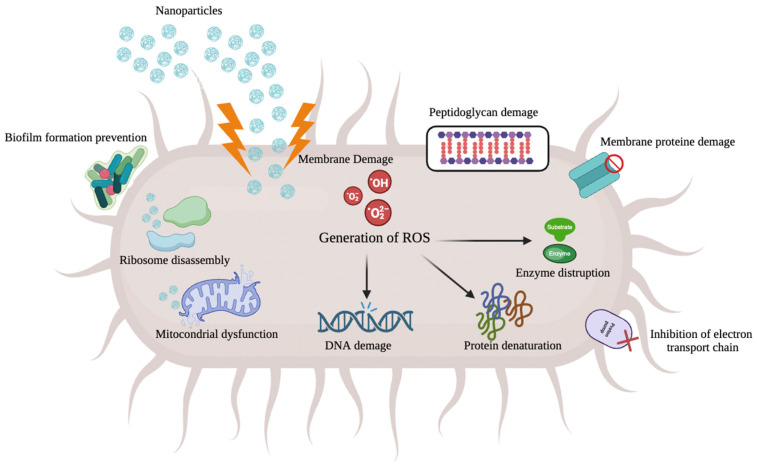
Common nanoparticle mechanisms of action in the bacterial host cell. Image created with BioRender.com (accessed on 10 August 2023).

Nanoparticles exhibit distinctive antimicrobial mechanisms that diverge from conventional treatments. The recent literature has reported on these mechanisms, including electrostatic interactions that disturb the cell membrane. This disturbance occurs when nanoparticles attach to the chondroid, leading to disruptions in cell respiration, division, and DNA replication [28]. Furthermore, nanoparticles can interfere with metal ion homeostasis, causing irreversible damage that inhibits microbial growth, and an excess of metal in nanoparticles can result in microbe death [29]. When nanoparticles adhere to the microorganism’s cell membrane, reactive oxygen species (ROS) are released, inducing oxidative stress and assaulting the organism. This action leads to further disruption of the cell membrane [30]. Nanoparticles can also form a protein-bound catalyzing agent called “carbonyl”, which initiates oxidative processes in the amino acid chain, leading to significant protein degradation, enzyme inactivation, and disruption of catalytic action [31]. Furthermore, due to their electrical properties, nanoparticles interact with nucleic acid molecules, affecting the replication of chromosomal and plasmid DNA and inhibiting signal transduction [32]. Reduced nanoparticle stability leads to weaker bonding and interfaces with other molecules, providing greater benefits [21]. The oral environment is highly intricate, facing consistent shear forces from activities such as tongue movement against the palate and oral mucosa against teeth. The shear rate depends on the viscosity of the lubrication and the level of the bolus [24]. Changes in shear stress can influence the characteristics of oral biofilm, affecting its morphology, thickness, and diversity [25]. High-shear bacterial infections in the oral cavity can be difficult to eradicate, necessitating novel antimicrobial strategies. One study by Yue Zhang et al. explored the use of a hydrogel hybrid containing antibiotics, showing effective drug release under shear stress [26]. According to the study by Hake et al., dental implant failure is often attributed to post-surgery osteomyelitis caused by three main types of bacteria: *Staphylococcus*, *Enterobacteriaceae*, and *Pseudomonas*. Among these, S. *aureus* and S. *epidermidis* are the primary pathogens responsible for osteomyelitis [33,34,35]. Numerous researchers have delved into the evaluation of the antimicrobial efficacy of nanoparticles against a broad spectrum of microbes. Despite numerous pieces of evidence, the complex functioning of their mode of action remains unclear. Using cutting-edge techniques such as high-resolution microscopy (AFM, FE-SEM, TEM, and XRD), spectroscopy (DLS, ESR spectroscopy, fluorescence spectroscopy, inductively coupled optical plasma emission spectroscopy, and UV-vis), and analysis of molecular and biochemical properties allowed us to reveal the mechanistic complexities behind the antimicrobial action of nanoparticles [36]. They exhibit their antimicrobial effects through four distinct mechanisms: (1) adhering to the cell wall and membrane surface, (2) infiltrating cells and causing damage to intracellular structures and biomolecules, (3) inducing oxidative stress through the generation of ROS and free radicals, and (4) influencing signal transduction pathways [37] (Figure 4).

Furthermore, nanoparticles also exert an influence on the human immune system, driving an inflammatory response that aids in pathogen suppression [38]. When microorganisms encounter AgNPs, they attach to the cell wall and membrane. The positive surface charge of nanoparticles plays a crucial role in this adhesion process. This positive charge creates an electrostatic attraction between the nanoparticles and the negatively charged cell wall of the microorganisms, making it easier for the nanoparticles to attach to cell membranes. This interaction leads to notable morphological changes, including shrinkage of the cytoplasm and detachment of the membrane, ultimately causing cell wall rupture [39]. Using TEM, it was observed that just a few minutes of contact with the nanoparticles destroys the cell wall of *E. coli* cells. The cell wall takes on a circumferential shape, and electron-dense pits appear at the sites of nanoparticle-induced damage. In addition to electrostatic attraction, the interaction of nanoparticles with sulfur-containing proteins in the cell wall causes irreversible changes, destroying the cell wall structure [40]. In some cases, after attaching to the cell wall, nanoparticles can penetrate cells, disrupting essential cellular functions [41]. The outer membrane of Gram-negative bacteria contains porins, which are involved in the internalization of nanoparticles into bacterial cells. Notably, altering *E. coli* porin protein expression in mutants made the cells more resistant to AgNP-based antibacterial therapy [42]. Once nanoparticles penetrate microbial cells, they interact with cellular structures and biomolecules such as proteins, lipids, and DNA, altering their structure and function. The interaction of nanoparticles with ribosomes leads to denaturation and subsequent inhibition of translation and protein synthesis [17]. Furthermore, they can interact with the functional groups of proteins, causing their deactivation. Nanoparticles induce DNA damage, including double-strand breaks. Indeed, mutations in crucial DNA repair genes (*mutY*, *mutS*, *mutM*, *mutT*, and *nth*) in *E. coli* make the mutant strains more susceptible to nanoparticle-based antibacterial therapy [43]. The notable antimicrobial capacity of nanoparticles is also due to their ability to generate ROS and free radicals such as hydrogen peroxide (H_2_O_2_), superoxide anion (O_2_^−^), hydroxyl radical (OH•), hypochlorous acid (HOCl), and singlet oxygen [44]. This leads to an escalation of oxidative stress within cells. Although the precise mechanism by which ROS mediates the antibacterial activity of nanoparticles remains unclear, it is established that the generation of ROS in bacterial cells causes cell death. Nanoparticles induce dysfunction in the respiratory electron transport chain by decoupling it from oxidative phosphorylation, achieved through the inhibition of respiratory chain enzymes. Elevated levels of ROS in cells also cause hyperoxidation of lipids, proteins, and DNA, altering cytoplasmic membrane structure and cellular metabolic pathways and promoting DNA double-strand breaks and mutagenesis [45]. Phosphorylation of various protein substrates serves as a crucial signal transmission mechanism in bacteria, playing a vital role in microbial growth and cellular activity. Nanoparticles are believed to influence cell signaling by dephosphorylating tyrosine residues on key bacterial peptide substrates, thereby hindering microbial growth. Treatment of *E. coli* and *Salmonella typhi* (*S. typhi*) with nanoparticles led to significant dephosphorylation of two peptides with relative masses of 150 and 110 kDa [46].

### Connections between Nanoparticle Activity and Their Physical and Chemical Characteristics

Several pieces of evidence have reported how the specific properties of these nanoparticles, such as size, shape, stability, and composition, play a fundamental role in influencing their antibacterial efficacy [47]. In a study by Lu et al., the impact of size on the antibacterial activity of AgNPs against bacteria associated with caries and periodontal diseases was investigated. AgNPs with sizes of 5, 15, and 55 nm were synthesized using chemical reduction with polyvinylpyrrolidone (PVP). Evaluation of their antibacterial activity against various microorganisms, including *E. coli*, *Fusobacterium nucleatum* (*F. nucleatum*), *Streptococcus mutans* (*S. mutans*), *Streptococcus sanguis* (*S. sanguis*), *Streptococcus mitis* (*S. mitis*), and *Aggregatibacter actinomycetemcomitans* (*A. actinomycetemcomitans)*, revealed that the 5 nm nanoparticles exhibited superior antibacterial effects. The nanoparticles completely inhibited microbial growth at concentrations ranging from 6 to 50 μg/mL. MIC values ranged between 6 and 50 μg/mL in response to treatment with 15 nm nanoparticles. Whereas doses of 100–200 μg/mL of 55 nm nanoparticles significantly impaired bacterial replication [48]. Azam et al. reported that CuNPs showed a significant inhibitory effect against Gram-negative and positive bacteria (*E. coli*, *Pseudomonas aeruginosa*, *Bacillus subtilis*, and *S. aureus*) compared to their larger counterparts. CuNPs of 20 nm inhibited microbial growth in the concentration range of 20–30 μg/mL. In contrast, a higher dose of 29 nm CuNPs was required to reduce the bacterial load (55–75 μg/m) [49]. It is now known that nanoparticles interact with bacteria in a shape-dependent manner. AgNPs interact with bacteria in a shape-dependent manner. Helmlinger et al. determined the antibacterial effect of AgNPs with different shapes, such as platelets, spheres, rods, and cubes. Silver nanoplatelets showed higher antibacterial activity against *S. aureus*, followed by spheres, rods, and cubes [50]. Furthermore, in a study by Cheong et al., sphere, disc, and triangular plate forms of AgNPs were chemically synthesized, and their antibacterial activity was evaluated against *E. coli*, *S. aureus*, and *P. aeruginosa*. The data reported higher antibacterial efficiency for spherical nanostructures, followed by discoidal and triangular nanoparticles [51]. In a study conducted by Babayevska et al., the antibacterial activity of ZnONPs, nanorods, particles, hierarchical structures, and tetrapods was evaluated against *E. coli* and *S. aureus*. The most effective structures were ZnONPs and nanotubes, significantly reducing the bacterial load to 500 µg/mL. Concentrations greater than 500 µg/mL of ZnO particles, hierarchical structures, and tetrapods were found to cause a slight reduction in bacterial replication [52]. While the size and shape of nanoparticles undoubtedly influence their activity, it is essential to consider the impact of other factors, including their chemical composition [53]. Nieto-Argüello et al. explored this further by synthesizing four distinct Ag/Au bimetallic nanoparticles with varying ratios, such as 20:80, 40:60, 60:40, and 80:20. The antibacterial activity of these nanoparticles was evaluated against methicillin-resistant *S. aureus* (MRSA) and multidrug-resistant *E. coli* (MDR-EC). The results revealed a relationship between the antibacterial activity and the composition of bimetallic nanoparticles. Notably, those with a higher percentage of Au demonstrated milder bacterial inhibition than those with a predominance of Ag. Nanoparticles displaying an Ag/Au ratio of 80:20 induced complete inhibition of MRSA growth and MDR-EC at concentrations of 4.5 and 7.9 µg/mL, respectively. In contrast, Ag/Au nanoparticles at a ratio of 20:80 significantly reduced the bacterial load of MRSA and MDR-EC at concentrations of 20.8 and 18.1 µg/mL, respectively [54]. Moreover, Bankier et al. assessed the antibacterial efficacy of tungsten carbide (WC), silver (Ag), and copper (Cu) nanoparticles, both individually and in various combinations (Ag40Cu10WC50, Ag10Cu40WC50, Ag20Cu30WC50, Ag20Cu50WC30, and Ag20Cu70WC10), against *S. aureus* and *P*. *aeruginosa*. The results underscored a notable enhancement in antimicrobial effects when nanoparticles featured combinations of metals, surpassing the efficacy of their individual counterparts. Specifically, nanoparticles comprising Ag10Cu40WC50, Ag20Cu30WC50, Ag20Cu50WC30, and Ag20Cu70WC10 (*w/w*%) exhibited complete inhibition of *P. aeruginosa* growth at a concentration of 0.05 *w*/*v*%. In contrast, higher concentrations of 0.25 *w*/*v*% of Ag40Cu10WC50 were required to significantly curb the growth of *S. aureus* [55]. The stability of nanoparticles plays a crucial role in determining their ultimate antibacterial activity. When synthesized nanoparticles exhibit low stability, they tend to aggregate and form larger particles. As demonstrated, larger nanoparticles generally show reduced antibacterial activity. The key factors influencing the stability of nanoparticles are charge and coating. Zeta potential has emerged as a critical parameter for evaluating the stability of nanoparticles. It has been established that AgNPs can be considered stable when their surface charge exceeds +30 mV or falls below −30 mV [56]. This is attributed to the repulsive interactions between the nanoparticles, which hinder their agglomeration. The zeta potential of nanoparticles is influenced by both the synthesis process and the choice of coating agent [57]. By carefully considering and manipulating these factors, researchers can ensure the stability of nanoparticles and optimize their antibacterial potential [58].

## 4. Nanoparticles in Endodontics

In modern endodontic treatment, the importance of combating bacterial contamination is of utmost significance in achieving successful outcomes. Nanoparticles have emerged as a promising solution to address this challenge, offering unique advantages over conventional materials and showcasing distinctive mechanisms of action (as shown in Table 1) [7,59]. Nowadays, nanotechnology can be employed to preserve and/or restore oral health, offering targeted treatments in cases of bacterial infections, dentin hypersensitivity, tissue regeneration, restoration of dentin integrity, and oral cancer [59]. Traditional sealants tend to exhibit short-lived antibacterial effects that decline over time, highlighting the need for more effective alternatives [60]. Today, a lot of different kinds of nanoparticles are available, and they can be classified according to their composition: metallic (copper, gold, silver), inorganic (zinc oxide, and titanium), polymeric (chitosan), functionalized with photosensitizers or drugs, and so on [61]. Other classification criteria are according to their mechanism of action, like electrical interactions with cell membranes and interruption of cell walls after damages caused by the production of ROS that lead to loss of cellular homeostasis and death [62]. Generally, nanoparticles’ extremely small size, elevated chemical reactivity, and high surface-to-mass ratio allow them to interact with biomolecules through the preparation of specific devices and biomaterials [61].

**Figure 5:**
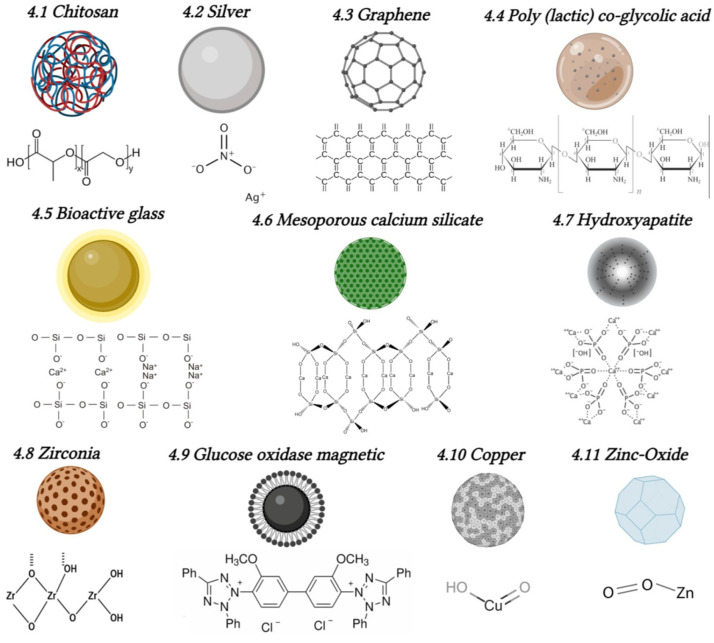
Schematic representation of the nanoparticles is described in the following paragraphs. Image created with BioRender.com (accessed on 10 November 2023).

The remarkable benefits and mechanisms of action exhibited by nanoparticles have sparked a rapid and substantial increase in their utilization in endodontics since their introduction. These versatile nanoparticles find applications in various aspects of endodontic therapy, including as components in irrigants, obturating materials, and intracanal medicaments (as depicted in Figure 3) [6,18,82,83,84]. In cases where biofilm formation poses challenges and eradicating bacteria becomes difficult, conventional antibiotics, whether administered topically or systemically, often prove ineffective. Consequently, the limitations of antibiofilm treatments in endodontic therapy have given rise to the development of advanced disinfection strategies. Among these strategies, researchers have turned their attention to the potential of various nanoparticles, including chitosan, AgNPs, graphene, poly (lactic) co-glycolic acid, bio-active glass nanoparticles, hydroxyapatite nanoparticles, mesoporous calcium silicate, and zirconia. These nanoparticles are being investigated as potential tools to overcome the limitations and address the challenges associated with biofilm-related issues [18,60]. Actually, within the realm of endodontic irrigants, NaOCl remains a popular choice, commonly utilized at concentrations ranging from 0.5% to 5.25% [22,23] and renowned for its tissue-dissolving properties and effective antimicrobial action [28,82]. NaOCl must be used with caution due to its potential for undesirable side effects. These side effects include the dissolution of the dentine matrix, weakening of the tooth structure, damage to periapical tissues, and persistent bacterial presence [30,31]. An alternative, chlorhexidine (CHX), is recommended as a milder endodontic sanitizer and is typically employed at a concentration of 2% [25]. Nevertheless, CHX also has limitations, such as its inability to dissolve necrotic tissue and its reduced efficacy against Gram-negative bacteria [32,85]. Another common chelating agent, ethylenediaminetetraacetic acid (EDTA), is utilized to remove the smear layer [18]. While EDTA has advantages, excessive usage can lead to dentine demineralization and weakening, especially when combined with NaOCl [6]. A combination of 2.5% NaOCl and 17% EDTA has shown a great effect against *Enterococcus faecalis* (*E. faecalis*), a common bacterial infection associated with endodontic infections [86]. The aforementioned limitations in conventional irrigation practices have fueled the growing interest in nanoparticle-based irrigants, with AgNPs standing out as particularly promising in this field. Overall, nanoparticles have opened up exciting possibilities for improving disinfection strategies and enhancing the overall effectiveness of endodontic treatments. Their continued exploration and development may hold the key to more successful outcomes and improved patient care in the field of endodontics. The following paragraphs will describe in detail the current evidence regarding the antimicrobial activity of nanoparticles and their potential use in endodontics (Figure 5).

### 4.1. Chitosan

Chitosan, a natural polysaccharide derived from deacetylated chitin, ranks as the second most abundant biopolymer and can undergo chemical modifications. Polymeric nanoparticles, particularly chitosan-based ones, have gained attention for their biocompatibility and remarkable antimicrobial properties [87]. Chitosan exhibits exceptional qualities in terms of antibacterial, antifungal, and antiviral action, demonstrating a minimum inhibitory concentration ranging from 18 to 5000 ppm, which positions it as a significant candidate in ongoing research [6]. In general medicine, chitosan is utilized as a wound dressing due to its ability to mimic the extracellular matrix and create an ideal environment for wound healing processes. In the context of endodontics, chitosan showcases its potent antimicrobial effect by effectively penetrating the intricate structures of root canals and dentinal tubules. It efficiently eliminates established microorganisms, with its sustained release property ensuring effectiveness for over three months. However, the effects of its antibacterial action are influenced by duration, concentration, and contact time. Its activity is inhibited by pulpal fragments and bovine serum albumin, whereas there are no effects from dental matrix and lipopolysaccharides (LPS) [6]. Barreras et al. demonstrated in an in vitro setting that the use of chitosan nanoparticles (CS-NPs) in conjunction with chlorhexidine successfully removed *E. faecalis* from the root canals [63]. A comparative in vitro study focused on the activity of CS-alone and CHX-loaded CS-NPs against *E. faecalis.* They concluded that the combination of CHX and CS-NPs was more effective at reducing the number of colony-forming units on agar plates despite nanoparticles alone [63].

Another study suggested that CS-NP action may depend on the state of bacteria, showing that planktonic bacteria were eliminated, whereas the biofilm persisted even after 72 h of treatment [88]. Application of CS-NPs strongly reduced the antibiofilm activity of other bacteria strains, as suggested by tests conducted on *Streptococcus oralis*, *Prevotella intermedia*, and *Actinomyces naeslundii* [70]. Research conducted by Carpio-Perochena et al. showed that treating bovine dentin sections with CS-NPs resulted in bacterial recolonization inhibition and reduced dentin smear, underlining the antibiofilm properties of this compound [89]. Chitosan’s low molecular weight enables it to penetrate the bacterial cell membrane, where it can bind to DNA, inhibiting transcription and mRNA synthesis. On the other hand, its high molecular weight is thought to interact with the negatively charged components of the bacterial cell wall, forming an impermeable layer that blocks transportation into the cell [90]. The negatively charged nature of chitosan enhances its ability to bind to the bacterial cell membrane (with a positive charge) through electrical magnetism, increasing permeability and eventually leading to the leakage of cytoplasmic contents and cell death [91]. Chitosan’s solubility in acidic environments leads to damage to cell membranes when its negative charges interact with positive amino groups, resulting in contact-mediated killing. Moreover, chitosan’s ability to chelate metals contributes to inhibiting microbial growth by reducing their activity through metal chelation [92].

The potential of chitosan as a root canal disinfectant was first explored by Kishen et al. in the realm of nanotechnology. Their study revealed that CS-NPs penetrate deep into the complexities of the root canal and dentinal tubules, effectively eliminating microorganisms over time, even up to three months [63].

CS-NPs can spread inside the root canal by applying an ultrasound of high intensity, preserving collagen through the action of bacteria, binding to dentine collagen, and offering protection against collagenase [93,94]. The exceptional properties of chitosan and its potential for controlled release and long-lasting antimicrobial effects make it a promising candidate in the field of endodontics, offering new possibilities for improving root canal disinfection and treatment outcomes.

Collectively, these studies affirm that CS-NPs hold significant potential for root canal disinfection. When used in conjunction with NaOCl, CS-NPs aid in deeper penetration into dentinal tubules [5]. However, conventional chelating agents like EDTA remain superior to CS-NPs in promoting sealer penetration, achieving a double rate of penetration [95].

### 4.2. Silver Nanoparticles

AgNPs have garnered considerable attention in dentistry, and their applications in endodontics are continuously expanding. Their unique properties render them highly effective in combating bacterial infections and promoting better treatment outcomes. Due to their small size and large surface area, AgNPs can easily penetrate the bacterial cell membrane, resulting in rapid bactericidal action through electrostatic interactions with superficial proteins, ROS production, DNA denaturation and disruption of ATP synthesis, inhibition of the transduction signal, and then death via apoptosis [96]. This makes them an excellent choice for enhancing disinfection protocols during endodontic procedures [56,97]. The biocompatibility of AgNPs, combined with their low bacterial resistance and minimal toxicity, makes them safe and well-tolerated in dental applications [56]. Biologically formed AgNPs have demonstrated remarkable antibacterial properties against *E. faecalis*, a challenging microorganism frequently encountered in endodontic infections [68,98,99]. These findings have spurred interest in leveraging AgNPs as a potent weapon against persistent bacterial biofilms within the root canal system. Nowadays, it is known that AgNPs have greater power of action and anti-biofilm activity if applied as a gel and not with a syringe because of the time- and contact-dependent nature of these nano-composts. The importance of surface charges and contact time of AgNPs also influences their antimicrobial properties: positively charged ones need less contact time and have a lower minimum inhibitory concentration than negatively or neutrally charged ones against *E. faecalis* [100]. AgNP antibiofilm activity against *E. faecalis* is weaker than chlorhexidine 2% after 5 min of treatments; they achieve the same results only after 15 min of treatment. However, NaOCl turned out to be the best material used as an antibacterial and in dissolving biofilm [101]. Polyvinyl alcohol (PVA)-coated AgNPs are good irrigants against *Candida albicans* (*C. albicans*), *Pseudomonas aeuroginosa* (*P. aeruginosa*), and *E. faecalis* [100]. Incorporating AgNPs into different aspects of endodontic treatment has shown promising results. For instance, AgNPs can be utilized as a substitute for NaOCl during the irrigation phase, providing an effective intracanal antimicrobial solution [102]. Researchers have also developed gutta-percha coated with AgNPs, creating an antimicrobial shutter for root canal obturation. This innovative approach not only seals the canal effectively but also prevents bacterial regrowth, improving the long-term success of the treatment [103,104]. Furthermore, AgNPs are being integrated as antibacterial additives into materials such as mineral trioxide aggregate (MTA). This enhancement has expanded the scope of MTA application, enabling its use in various procedures such as pulp-capping, apexification, and sealing perforations in teeth [103,104]. AgNPs have also been encapsulated in silica, and this combination was very strategic because it showed major and longer antimicrobial activity, resulting in minimal biofilm regrowth after 7 days of treatment instead of AgNPs alone, where regrowth occurred after 2 days [105]. AgNPs have demonstrated the potential to improve the outcomes of these crucial endodontic procedures by providing an extra layer of antimicrobial protection. An example highlighting AgNP efficacy was observed in a study by Sadek et al., wherein radicular human dentin specimens were inoculated with *E. faecalis* to form 3-week-old biofilms. AgNPs at a concentration of 0.02% were then introduced as antimicrobials, resulting in significant inhibition of bacterial growth [86]. The same concentration of AgNPs also had better results than calcium hydroxide and syringe irrigation, with a higher concentration of AgNPs (0.1%) when applied in the form of gel for 7 days. The final result was the destruction of the biofilm [106].

These findings indicate the potential of AgNPs for eradicating established biofilms and preventing reinfection within the root canal. This characteristic is conserved even when they are incorporated into biomaterials. Their silver ion ability against bacteria allows them to be used in combination with antibiotics. In combination with cefazolin (CEF), mupirocin (MUP), or gentamicin (GEN), they showed greater efficacy against *Staphylococcus aureus* (*S. aureus*), *P. aeruginosa*, and *Escherichia coli* (*E. coli*) [107]. The antimicrobial and anti-biofilm activity of AgNPs has also been confirmed when they have been coated with polyvinyl alcohol and farnesol in a study by Chavez-Andrade et al., and they confirmed AgNPs properties against *E. faecalis*, *C. albicans,* and *P. aeruginosa*, promoting their use in endodontic treatments [41]. However, bacteria Gram-negative like *E. coli* and *P. aeruginosa* can become resistant to AgNPs producing flagellin, a protein that promotes the aggregation of NPs after repeated exposures, inhibiting their action [108]. This interferes with the release of silver ions by decreasing the antimicrobial effect of nanoparticles. At the same time, AgNP agglomerations lead to a major cytotoxic effect, more abundant production of ROS, and then a pro-inflammatory effect. To prevent this, it is possible to use stabilizers like imidazole [109]. Indeed, AgNPs in combination with imidazole show less cytotoxicity, as reported in the Abbaszadegan et al. study [66]. Recently, a mix of silver ions (0.003%) in citric acid (4.846%) has been tested as an innovative cleaning and disinfecting material, showing good antibacterial activity with low pH-related toxicity; however, further details are needed because at concentrations higher than 5%, the mixture was harmful [110]. The same anti-microbial and anti-biofilm behavior of AgNPs has been confirmed when they have been associated with graphene oxide: the reduction in the number of bacterial colonies was influenced by nanoparticle dimensions and contact time [111]. Hiraishi et al. demonstrated that silver ions in the form of silver diamine fluoride were completely able to eradicate the microbial layer after one hour of treatment [6]. The best antimicrobial effect has been reached in an in vitro study made by Charannya et al. employing a combination of 15 µg/mL AgNPs and 2% CHX solution; this mix showed great efficacy against *E. faecalis*, *Klebsiella pneumoniae,* and *C. albicans* [112]. The versatile applications of AgNPs in endodontics, coupled with their potent antimicrobial properties and biocompatibility, offer promising avenues for improving root canal disinfection and enhancing the overall success of endodontic treatments. For this purpose, a fascinating new silver-based colloidal material has recently been developed: ARGITOS is an odorless and colorless solution obtained with green chemistry approaches, containing silver particles (1–2 nm in size), distillate, and sodium peroxide (a stabilizer). This innovative solution has shown strong antibacterial power against *E. faecalis* [113]. Overall, from the reported evidence, it is demonstrated that although AgNPs show a large number of advantages and positive aspects, there are also side aspects to be taken into consideration, such as toxic effects on mammalian cells and staining and blackening of dentin, which induce discoloration [114].

### 4.3. Graphene

Graphene-based nanomaterials (GBNs) have sparked significant interest within the dental field due to their distinctive properties and potential applications. Due to their excellent physicomechanical characteristics, stability, biocompatibility, biodegradability, electrical conductivity, and antimicrobial properties, good mechanical properties are useful in the diagnosis and treatment of dental diseases in antimicrobial materials and coatings [115]. GBN was found to be highly effective in preventing bacterial colonization on tooth surfaces, including root canal walls and dental implants [116]. These properties reduce the risk of post-treatment infections and improve the success rates of endodontic procedures. Furthermore, GBs are promising in the development of advanced drug delivery systems in endodontics. Their large surface area and ability to functionalize with various molecules make them ideal candidates for delivering therapeutic agents directly to the site of infection or inflammation [117].

This targeted drug delivery approach can enhance the efficacy of treatment while minimizing side effects. Additionally, GBNs’ unique physicochemical properties have the potential to improve the mechanical and antimicrobial characteristics of dental materials. Incorporating GBNs in dental restorative materials, such as composites and cement, could lead to stronger, more durable, and infection-resistant dental restorations [118]. Graphene can also be incorporated into bioactive cement like Biodentine, accelerating the hydration process and reducing induction time [119]. GBNs are available in several forms, including reduced graphene oxide (eGO), graphene oxide (monolayer up to several layers), and graphene nanocards (GNS). Due to their high surface sensitivity and ability to interact with biomolecules, GBNs are particularly suitable for the development of biosensors that detect specific oral pathogens and biomarkers related to oral health conditions [117]. Additionally, GBNs exhibit promise in tissue engineering applications, particularly in regenerating dental pulp and periapical tissues. As a coating on titanium implants, graphene has shown properties of osseointegration, tissue healing acceleration, and inhibition of microbial growth [120]. AgNP-coated graphene, instead, has reduced toxic effects on bone and soft tissues, keeping its antibacterial properties unaltered compared to 3% NaOCl [5,121]. It is vital to acknowledge that, like any emerging technology, safety considerations are paramount. Further research is essential to establish the biocompatibility and long-term effects of GBNs in oral tissues. Furthermore, standardization and regulation of GBN usage in dental materials and therapies are imperative to ensure their safe and effective integration into clinical practice. As research in this field advances, GBNs are likely to become integral components of advanced dental materials and therapeutic strategies, ultimately benefiting patients and advancing oral healthcare practices.

### 4.4. Poly (Lactic) Co-Glycolic Acid

In recent research, poly (lactic) co-glycolic acid nanoparticles (PLGA NPs) loaded with a photosensitizer called methylene blue have shown promising results in improving endodontic disinfection. Methylene blue is a photoactive medicine that becomes highly reactive when exposed to light of a specific wavelength. This property allows it to generate reactive oxygen species (ROS) when illuminated, leading to the destruction of microbial cells. In a study conducted by researchers Pagonis et al., the utilization of PLGA NPs loaded with methylene blue was employed within root canals afflicted by *E. faecalis*, a prominent bacterial pathogen implicated in root canal infections [122]. Once the nanoparticles were applied, a low-power laser with the appropriate wavelength was directed into the root canal, activating the methylene blue within the nanoparticles. The photoactivation of methylene blue-loaded PLGA nanoparticles resulted in the generation of ROS, which had a potent bactericidal effect on the *E. faecalis* bacteria present in the root canal system. The ROS attacks the bacterial cell membranes, proteins, and nucleic acids, leading to bacterial cell death. The combination of PLGA nanoparticles and photoactive medicine offers several advantages in endodontics. Firstly, the nanoparticles can be designed to specifically target infected areas within the root canal, ensuring a more focused and effective antimicrobial action. Secondly, the use of a low-power laser for photoactivation minimizes any potential adverse effects on surrounding tissues. Moreover, this approach has the potential to address the challenges of conventional endodontic disinfection methods, especially when dealing with persistent or antibiotic-resistant bacterial infections. In this pursuit, various other photosensitizers have been explored. For instance, rose Bengal-conjugated chitosan nanoparticles showed significant antimicrobial potential in a study conducted by Shrestha et al., low cytotoxicity levels, antibiofilm properties, and inactivation of endotoxins [70]. These nanoparticles exhibited a 50–65% reduction in *E. faecalis* planktonic rates. When coupled with photoactivation, these nanoparticles exhibited complete microbial eradication after 24 h. This strategy also showcased improved dentine properties, attributed to chitosan nanoparticles binding to collagenase, thus inhibiting collagenolytic activity [70,123,124]. Also, methylene blue-conjugated chitosan nanoparticles had stronger antibacterial action against *E. faecalis* in infected root canals if compared with chitosan nanoparticles or methylene blue alone [125,126]. Another attractive photosynthesizer that has gained attention is indocyanine green because, in combination with AgNPs, it produces a 99.12% reduction in *E. faecalis* colony-forming units (CFU). This result is higher than the application of nanoparticles and indocyanine green alone [82]. By incorporating PLGA NPs and photoactive medicines, endodontists can enhance the success rates of root canal treatments and reduce the risk of treatment failure due to persistent microbial presence [122].

These recent studies collectively contribute to the exploration of innovative disinfection strategies, particularly the synergy of functionalized nanoparticles and photodynamic therapy, which have the potential to ameliorate dentine’s physical properties. Despite prevailing limitations such as the potential for aggregate formation and the challenge of infiltrating complex anatomical spaces, future studies may refine and optimize these novel strategies, ushering in an era of innovative disinfection modalities [113,114].

### 4.5. Bioactive Glass Nanoparticles

Ensuring successful root canal therapy relies heavily on effectively sealing the root canal system. Root canal sealers play a crucial role in this process, as they are responsible for filling voids in the root canal, eliminating residual bacteria, and sealing off irregularities, including small lateral canals and isthmuses [127,128]. Among the biomaterials commonly used for dental caries repair and pulp capping, conventional calcium hydroxide-based materials, particularly mineral trioxide aggregate (MTA), have been a popular choice [129]. However, MTA has its limitations, such as its long solidification time, poor handling properties, potential toxicity, and high cost [130]. As a result, there is a growing demand for low-cost alternative biomaterials for dentin and pulp tissue repair. Researchers Vichery and Nedelec, among others, have explored the use of mesoporous bioactive glass (BG) nanoparticles to enhance both the antimicrobial and tissue regeneration potentials [131]. This new material can also be employed as an alternative dentin sealer owing to its ability to directly adhere to the bone. New bone tissue can be formed on bioactive glass, facilitated with the adhesion of ions to the silica surface, resulting in the formation of hydroxyl carbonate apatite (HCA). Hydroxyapatite is then covered with osteogenic cells, and a coated bioactive glass is obtained. In the end, crystallization leads to new bone tissue [132]. Fabricated through the widely employed sol–gel process, these nanoparticles contribute to biomedical applications [133]. One of the critical challenges during dentin and pulp tissue regeneration is the uncontrolled growth of bacterial colonization, leading to an acidic pH environment that can lead to tooth decay. The detrimental influence of this acidic pH extends to the physicochemical properties of regenerative sealing materials, the viability and differentiation of precursor cells, and tissue-specific cells [134].

Therefore, the development of bioactive dental materials capable of neutralizing acidic pH while maintaining optimal physicochemical properties and cellular activity is essential. Nanocomposites have shown promise in restoring and regenerating mineralized dental tissues, providing increased mechanical strength and osteoconductivity [135]. Among these compounds, bioactive glass-based nanoparticles (BG-NPs) are considered a cutting-edge biomaterial with pro-angiogenic and anti-inflammatory properties [64]. These nanoparticles primarily consist of SiO_2_, Na_2_O, and P_2_O_5_ at specific concentrations, forming the principal components of BG-NPs, which typically range in size from 20 to 60 nm [136]. BG-NPs offer various benefits, like alkaline pH through the release of ions in water; osmotic effects with an increase in osmotic pressure, which, if greater than 1%, causes bacterial death; and enamel remineralization after calcium-phosphate precipitation. The integration of BG-NPs in dental materials holds significant potential for advancing regenerative endodontic therapies and improving long-term treatment outcomes. With this aim, Correira et al. have tested three different bioactive glass compositions produced using the sol–gel method to understand which one was the best for removing pathogens responsible for tooth reinfections and to find long-term treatments [137]. The three compositions differed in terms of the content of SiO_2_, CaO, MgO, and CuO. In this study, it has emerged that two BG compositions can inhibit the growth of *E. faecalis* after 48 h of incubation, whereas all samples can reduce *C. albicans* survival [137]. By addressing the challenges associated with conventional biomaterials and enhancing their properties through nanotechnology, researchers aim to pave the way for more effective and biocompatible solutions in endodontics and dental tissue regeneration.

### 4.6. Mesoporous Calcium Silicate

Mesoporous calcium silicate nanoparticles (MCSNs) have emerged as advanced biomaterials with remarkable properties that make them highly attractive for dental applications. One of the notable features of MCSNs is their ability to promote the formation of apatite mineralization and the controlled release of drugs in the apical root canal of teeth, making them valuable in endodontic therapies [106]. Additionally, MCSNs have been shown to support osteogenic differentiation of stem cells in vitro, indicating their potential for promoting tissue regeneration and repair. MCSNs are characterized by their size, typically ranging from 80 to 100 nm, which provides them with a high specific surface area and pore volume ratio [68]. Researchers like Fan et al. have explored the functionalization of MCSNs with chlorhexidine to evaluate their antimicrobial properties, drug release profile, and effects on cell proliferation and in vitro mineralization properties [74]. Huang et al. have investigated the possibility of using these nanoparticles for drug delivery, especially antibiotics like gentamicin, to potentiate antimicrobial properties [138]. Such studies pave the way for utilizing MCSNs as antimicrobial agents and drug delivery vehicles in endodontic treatments. Another exciting avenue of research involves combining MCSNs with AgNPs and zinc nanoparticles (ZnNPs). Jie Zhu et al. investigated the antimicrobial activity of MCSNs loaded with AgNPs and discovered that the combination of Ag and Zn with MCSNs (Ag-Zn-MCSNs) using a template process could lead to a new disinfectant for root canals and dentinal tubules [139]. This combination exhibits good antibiofilm efficacy, biocompatibility, osteogenic properties, minimal cytotoxicity, sustained ion release, dentinal tubule infiltration, and injection [90]. Furthermore, Diya Leng et al. conducted a study on the antibiofilm activity and mechanism of action of Ag/Zn-MCSNs at different concentrations of Ag and Zn [140]. Additionally, Ag-MCSNs release silver ions, inhibiting *E. faecalis* colonization [141,142]. These findings shed light on the potential of MCSNs as a promising approach to combating bacterial biofilms in endodontics, addressing one of the major challenges in root canal treatments. As researchers delve deeper into the properties and potential applications of MCSNs, the future of dental biomaterials and regenerative therapies looks promising. The versatility of MCSNs in drug delivery, antimicrobial action, and tissue regeneration opens up new possibilities for enhancing dental treatments and improving patient outcomes. While the application of MCSNs in dentistry shows great potential, more comprehensive studies and clinical trials are needed to fully understand their safety and long-term effects.

### 4.7. Hydroxyapatite Nanoparticles

Nano-hydroxyapatite (HAp) has gained significant attention in the field of oral care products for its potential to treat oral hypersensitivity and promote enamel remineralization. This bioactive and biocompatible material shares a similar chemical composition with the apatite crystals found in human enamel and is capable of reducing local or systemic inflammatory reactions [142,143,144]. Numerous in vitro and in situ studies have documented its remarkable remineralization, preventive capabilities in oral treatments and dental caries, reduction in dentinal hypersensitivity, and restoration of tooth deformities [145,146,147]. HAp has demonstrated a strong ability to adhere to tooth surfaces, effectively absorbing plaque components and bacteria, further supporting its potential as an effective oral care ingredient [148,149,150]. The main function of nano-hydroxyapatite is to integrate the dentinal tubules and seal their openings, preventing the exposure of nerves to external stimuli [68]. Despite the promising results, there have been discussions regarding the regulatory and safety concerns surrounding the use of nano-hydroxyapatite in oral care products. The European Scientific Committee on Consumer Safety (SCCS) has raised questions about its usage. However, Japan (in 1993) and Canada (in 2015) approved HAp as an active agent in oral care products, showcasing its non-inferiority and equivalence to fluoride [150,151]. A study conducted by Coelho et al. addressed the concerns raised by the SCCS and concluded that specific hydroxyapatite nanoparticles are cytocompatible, as they do not alter the normal activity of cells. This supports their safe use in various oral care products [152]. HAp has shown promising results in reducing dentin hypersensitivity and can be found in various dentifrices and mouthwash solutions. Its mode of action involves remineralizing demineralized enamel surfaces through the precipitation of calcium and phosphate, which are provided by hydroxyapatite [153]. The addition of HAp to the polymeric matrix improves its mechanical properties. HAp added to chitosan scaffolds upregulates the expression of some genes like Runx2, ALP Smad1, BMP-2/4, collagen I, integrin, and myosins. This leads to an increase in bone marrow stem cell proliferation, as suggested in the Liu et al. study [154]. Due to its exceptional biocompatibility, HAp can also be applied as a periapical healing material [57]. As research in nanotechnology and biomaterials continues to evolve, HAp holds tremendous potential for revolutionizing oral care and addressing various dental concerns thanks to its better properties (smaller contact angles and a greater number of surface atoms) in contrast with conventional microsized materials.

### 4.8. Zirconia

Zirconium is a biocompatible and non-toxic metal, making it a preferred material for orthopedic and dental implantation. Its excellent mechanical properties and remarkable corrosion resistance further enhance its suitability for medical applications. It has been proposed as a substitute for titanium implants because of its biocompatibility, lower risk of early plaque formation, lesser cytotoxicity, and good osseointegration ability [155]. With the increasing concern over antimicrobial resistance, nanoparticles have emerged as an efficient alternative for combating various antimicrobial-resistant bacteria. Metal oxides like ZnO, TiO_2_, Fe_2_O_3_, and CuO_3_ are known for their significant antimicrobial properties. A study conducted by Khan et al. involved the synthesis of cubic ZrO_2_NPs and their biofunctionalization using L-glutamic acid as a ligand. These nanoparticles were tested for their antimicrobial activity against bacterial pathogens, including *R. muciliginosa*, *R. dentocariosa*, *S. mitis,* and *S. mutans* [156]. The results demonstrated the potential of ZrO_2_NPs as effective agents against these bacterial strains and also in the treatment of oral illnesses, eradication of the smear layer, and biofilm development [5]. In dentistry, zirconia is recognized as a chemical oxide widely used for its optical and metallic properties, which closely resemble those of natural teeth. Zirconia has been found to effectively eliminate bacterial colonization, causing low cytotoxic effects due to its insolubility in water [157]. Zirconia-based nanoparticles have shown high potency against specific microorganisms, such as *E. faecalis*, and are increasingly utilized as antimicrobial agents in endodontic treatments [78,133]. The use of zirconia and zirconia-based nanoparticles in dental applications offers promising prospects for combating bacterial infections and improving overall oral health. They can also be mixed with polymers or deposited onto biomaterial surfaces, enhancing remineralization and combating caries caused by oral bacteria strains [158]. Nowadays, it is possible to synthesize ZrO_2_NPs with a green chemical approach, using garlic and ginger together with zirconium. This combination has shown potent antibacterial and antibiofilm activity against *S. aureus* [159]. Despite the numerous advantages deriving from the use of zirconia nanoparticles, an in vitro study by Nemec et al. has investigated the response of *Porphyromonas gengivalis* (*P. gengivalis*, a very common Gram-negative bacteria in the oral cavity that is responsible for periodontal diseases) against titanium and zirconia nanoparticles (<100 nm). These compounds have been tested on human gingival mesenchymal stromal cells with different concentrations of nanoparticles for 24 h. Both nanoparticles have stimulated a significant expression of IL-8 (a pro-inflammatory mediator in gingivitis) induced by *P. gengivalis*, suggesting that nanoparticles can be potential risk factors for peri-implant diseases. So, further studies are needed to better understand the safety profile and success rate of the use of these materials [160].

### 4.9. Glucose Oxidase Magnetic Nanoparticles

Magnetic nanoparticles (MNPs) in combination with glucose oxidase (GOx) are emerging as new strategies to eradicate bacterial/fungal endodontic infections [161]. GOx-modified MNPs (GMNPs) exhibit antimicrobial activity against *E. faecalis* and *C. albicans* through the production of ROS and nutrient deficit [80]. Their small size, strong magnetism, biocompatibility, and cell membrane penetration capability make them highly attractive for antibacterial and antifungal therapy. As alternatives to conventional disinfectants such as Ca(OH)_2_, NaOCl, and CHX, GMNPs hold significant promise due to their ability to overcome the limitations of traditional approaches [162]. The abilities of GMNPs against *E. faecalis* and *C. albicans* were tested in a study conducted by Ji et al., which showed a reduction in the number of bacterial and fungal colonies after MNP exposure [163]. This behavior occurred mainly when the concentration of GMNPs increased from 0.1 to 1.0 mg/mL. To better understand the antibacterial and antifungal properties of GMNPs, inhibition rates were calculated, showing that >80% of *E. faecalis* were killed by 1 mg/mL of GMNPs, and the same concentration was involved in *C. albicans* death with a rate of 98.84%. The mechanism of action of GMNPs is similar to that of other nanoparticles because they all produce ROS, damage cell walls, and cause death after starvation of nutrients. Scientists have also evaluated the antibiofilm activity of GMNPs by applying them with and without an external magnetic field to homogeneous biofilm formed after culturing *C. albicans* and *E. faecalis* for 48 h, finding that MNPs efficiently attach to biofilms and easily penetrate through channels. GOx catalyzed the oxidation of glucose, and then reactive oxygen species like H_2_O_2_ and OH- were produced. They destroyed the biofilm matrix, confirming the antibiofilm activity of GMNPs [163]. Overall, the excellent anti-bacterial properties and ability of GMNPs to destroy dense matrix biofilm suggest that they can be successfully utilized against bacterial/fungal endodontic infections [81].

### 4.10. Copper Nanoparticles

Copper nanoparticles exhibit potent anti-inflammatory and antimicrobial properties, offering versatility in combination with other materials due to their biocompatibility. CuNPs leverage their positive charges to attract negative carboxyl groups of bacterial lipoproteins, and then ions accumulate in bacterial membranes, changing their permeability [164]. This alteration causes the degradation of proteins and DNA and the production of ROS, leading to cell death [165]. In endodontic applications, the incorporation of CuNPs into pastes has shown promise in inhibiting the growth and replication of root canal-associated oral microbes responsible for biofilm formation [166]. This utilization enhances the prevention and treatment of biofilm-related endodontic infections [167]. Furthermore, the amalgamation of CuNPs with titanium has been explored, revealing improved antimicrobial properties against oral microorganisms and enhanced biocompatibility compared to using copper or titanium alone. Despite encouraging in vitro findings, transitioning these advantages to clinical settings presents challenges. Subsequent research endeavors are essential to comprehensively elucidate the mechanisms and characteristics of CuNPs, aiding in their effective translation to clinical applications.

In their study, Lewis Oscar et al. demonstrated that CuNPs can reduce the biofilm formation of *P. aeruginosa*, offering a valid strategy to overcome the problem related to antibiotic resistance [168].

### 4.11. Zinc Oxide Nanoparticles

Zinc oxide nanoparticles (ZnONPs) are metal oxide nanoparticles with a broad spectrum of applications in biomedicine and dentistry, thanks to their exceptional anti-microbial, regenerative, and mechanical properties. They are particularly used as antimicrobials and antifungals against oral microflora and pathogens [169]. Other positive aspects of these nanoparticles are their good optical, magnetic, and electrical properties that allow them to be used in drug delivery systems and dentinal lesion remineralization, as evidenced in the Pushpalatha et al. study [170]. ZnONPs have also been investigated in association with anti-microbial agents, thanks to their drug delivery ability. It has been demonstrated that the acid environment of the dentinal lesions causes the degradation of nanoparticles, facilitating the release of ions and substances bound to them. A previous study suggested that the association of ZnONPs with CHX and Ca(OH)_2_ promoted dentinal remineralization and had stronger activity against *E. faecalis*. This combination also showed a better ability to penetrate dentinal tubules [159]. Furthermore, gutta-percha coated with zinc oxide nanoparticles was effective in counteracting the growth of *S. aureus* and *E. faecalis*, especially as sealants, reducing the risk of reinfection after treatment [171].

## 5. Discussion and Conclusions

The application of nanoparticles in dentistry, particularly in endodontics, holds great promise for advancing treatment options. The ability of nanoparticles to penetrate and disrupt biofilms, their antimicrobial properties, and their biocompatibility make them valuable tools in combating oral infections and promoting dental health. One of the significant advantages of nanoparticles is their potential to target and eradicate bacteria within complex anatomical structures like root canals but also their ability to act only in the area of interest without damaging the surrounding tissues. Thanks to their nanometric dimensions, they can be suitably designed to better reach their targets compared to conventional sealers or irrigants. Traditional mechanical instrumentation and irrigation alone may not effectively reach all areas, leaving residual biofilms and bacteria that can compromise treatment outcomes [172]. However, when nanoparticles are incorporated into irrigation solutions or intracanal medicaments, they can infiltrate the dentinal tubules and intricacies of the root canal system, ensuring a more thorough and long-lasting antimicrobial effect [70,82,84]. In addition to their role in disinfection, nanoparticles are also being explored for their regenerative properties. Mesoporous calcium silicate nanoparticles (MCSNs) have demonstrated the ability to promote apatite mineralization and controlled drug release in root canals, thereby facilitating tissue regeneration and enhancing the healing process [106]. Graphene-based nanomaterials (GBNs) are being investigated for their potential in enamel remineralization and hypersensitivity treatment [117]. Zinc oxide nanoparticles represent a good chance for drug delivery. GM NPs can eradicate persistent bacterial/fungal endodontic infections, showing broad-spectrum antimicrobial potential. Anti-biofilm ability also helps to avoid re-infection, ensuring longer-term treatments. Recently, the use of poly (lactic) co-glycolic acid nanoparticles (PLGA NPs) loaded with a photosynthesizer has offered numerous advantages since this medicine increases its ability to produce ROS when exposed to a specific wavelength, which can be translated into a more powerful bactericidal effect of nanoparticles. So, the combination of functionalized nanoparticles with photodynamic therapy has the advantage of minimizing any potential adverse effects on surrounding tissues. A very promising alternative seems to be the use of bioactive glass nanoparticles, thanks to their ability to adhere directly to the bone, also favoring the tissue regeneration process. These advancements show the versatility of nanoparticles in addressing various dental issues. Despite the promising results, it is essential to investigate safety concerns and establish clear guidelines for the use of nanoparticles in dentistry [173]. Toxicity and potential long-term effects must be thoroughly evaluated to ensure patient safety and optimize the benefits of nanoparticle-based treatments [174]. It is very important to take into consideration that the use of nanoparticles must limit the occurrence of side effects like toxic effects on mammalian cells and staining and blackening of dentin, inducing discoloration [175]. In conclusion, nanoparticles have emerged as valuable tools in endodontic therapy and dentistry as a whole. Their unique properties enable effective biofilm disruption, enhanced disinfection, and even tissue regeneration. Continued research and development in this field will undoubtedly lead to further innovations and improvements in dental care. However, caution must be exercised to ensure the safe and responsible use of nanoparticles in clinical practice. By embracing these advancements with careful consideration, dental professionals can provide more effective and personalized treatment options, ultimately benefiting the oral health and well-being of patients.

## Figures and Tables

**Table 1:** Different classes of nanoparticles and their application for endodontic treatment.

Sr. No.	Nanoparticles	Application	References
1.	Graphene	Antimicrobial properties of graphene oxide nanoparticles against common pathogens like *S. mutans*	[4,5]
2.	Chitosan	They have excellent antimicrobial, antifungal, and antiviral activity based on electrostatic interaction, which leads to cell membrane disruption	[6,18,63]
3.	Combination of chitosan nanoparticles and ZnONPs	Good antimicrobial activity against *E. faecalis*	[64]
4.	Poly (lactic) co-glycolic acid	Conjugated with photoactive drugs and used for the eradication of microorganisms from endodontic canals	[5]
5.	Poly (vinyl alcohol) (PVA)-coated AgNPs	Efficacious against *P. aeruginosa*, *C. albicans,* and *E. faecalis*	[65,66]
6.	Silver nanoparticles (AgNPs)	AgNPs were observed to have antimicrobial and antifungal activity efficient against *E. faecalis*	[67,68]
7.	Zinc oxide nanoparticles (ZnONPs)	ZnONPs have the ability to remove the planktonic *E. faecalis* and are able to disrupt the biofilm matrix	[69]
8.	Photoactivated rose Bengal-conjugated chitosan nanoparticles	Inactivate endotoxins, in the presence of tissue inhibitors and functionalized nanoparticles showed a 50–65% reduction in planktonic *E. faecalis*	[70]
9.	Calcium hydroxide nanoparticles	These nanoparticles improve the depth of penetration, increase surface area contact with pathogens, have superior solubility, and have greater antimicrobial activity	[71,72]
10.	Chitosan and poly(lactic-co-glycolic) acid (PLGA)	Act as potential intracanal antibiotic delivery agents and possess good antimicrobial effects over 2 weeks	[73]
11.	Porous calcium silicate and bioactive glass nanoparticles	Calcium silicate compounds are bioactive, biocompatible, and osteogenic because of their internal porous structures as well as their potential to act as drug carriers	[50]
12.	Propolis-loaded PLGA nanoparticles	Antimicrobial activity against *E. faecalis*, *S. mutans,* and *C. albicans*. One of the applications was when doxycycline-functionalized polymP-n active nanoparticles were found to occlude dentinal tubules and exert against *E. faecalis*	[74]
13.	Mesoporous calcium silicate	Drug delivery, antibacterial efficiencies, injectability, apatite mineralization, and osteo-stimulation	[75]
14.	Hydroxyapatite nanoparticles	HAp integrates inside the dental tubules and seals the opening, helping prevent exposure to nerves to obnoxious external stimuli; therefore, they are used for decreasing dentin hypersensitivity	[76]
15.	Iron compound (FeOx)	Helps in antibiotics for the removal of endodontic biofilms	[77]
16.	Zirconia	Successfully used in the field of dentistry due to its optical and metallic properties similar to those of a tooth, and it also has antimicrobial effects	[64]
17.	TiO_2_ nanoparticles	Used as an effective antifungal for fluconazole-resistant strains	[78,79]
18.	CuO nanoparticles	Nanoparticles are active against Gram-positive and Gram-negative bacteria; they can cross the bacterial cell membrane and damage vital enzymes of bacteria and also possess antifungal activity	[57,75]
19.	GOx-modified MNPs (GMNPs)	GOx-modified MNPs (GMNPs) exhibit antimicrobial activity against *E. faecalis* and *C. albicans* The ability of GMNPs to destroy dense matrix biofilm suggests that they can be successfully utilized against bacterial/fungal endodontic infections	[80,81]

## Data Availability

The data presented in this study are available herein.
